# Beyond the Written Reflection: A Systematic Review and Qualitative Synthesis of Creative Approaches to Reflective Learning Amongst Medical Students

**DOI:** 10.5334/pme.914

**Published:** 2023-09-11

**Authors:** William MacAskill, Weng Joe Chua, Hannah Woodall, Janani Pinidiyapathirage

**Affiliations:** 1 Griffith University, Rural Clinical School, Toowoomba, QLD 4350, AU; 2 Rural Medical Education Australia, Toowoomba, QLD 4350, AU

## Abstract

**Introduction:**

In medical curricula, reflective learning (RL) mostly consists of writing and small-group discussion, yet accommodating diverse learning preferences is a key factor in developing lifelong reflective practitioners. Medical education uses a number of creative approaches to RL which cater to more diverse learning preferences; however, the overarching benefits of creative RL to students’ development is unknown. To understand how creative RL approaches contribute to students’ holistic development we performed a qualitative systematic review and synthesis.

**Methods:**

Systematic searches of PubMed, PsycINFO, and EMBASE databases identified 4986 unique records, with 15 studies meeting inclusion criteria. Included studies specifically assessed the impact of RL on medical students and utilized creative approaches to RL. Creative approaches were defined as those not predominantly focused on reflective writing or group discussion. Studies were appraised using the Critical Appraisal Skills Programme and the Checklist for Quasi-Experimental Studies.

**Results:**

We identified five distinctive RL methods: viewing, performing, creating, imagining, and mind-body. Thematic analysis generated three themes: building and maintaining relationships, personal development, and sense of belonging. These themes incorporated eight sub-themes: recognizing multiple perspectives, empathizing with others, two-way communication skills, patient centered care, processing thoughts and emotions, self-care, interacting positively with peers, and developing trust and commonality.

**Discussion:**

Creative RL approaches may foster students’ sense of belonging and support interpersonal skills and personal development. In addition, creative RL activities may contribute to medical graduate’s holistic development, while providing opportunities to address diverse student needs using innovative, non-conventional methods.

## Introduction

In medical education, reflective learning (RL) supports students to recall and re-evaluate their experiences and attend to their feelings [1]. The process is expected to create positive changes in future behaviors by reconstructing, reorganizing, and adding meaning to experiences [2,3]. RL links professional knowledge, practical competences, and professional activity [4], and supports deeper self-regulated learning, professional identity development [5], and therapeutic doctor-patient relationships [6]. RL is associated with increased medical student self-awareness, decreased bias, improved communication [7], empathy [8], professionalism, clinical reasoning and management of complex patients [6,9]. The methodological options for RL are broad and can be undertaken during (reflection-in-action) or after (reflection-on-action) practice [10]. RL can be structured or unstructured; regular or periodical; based upon writing or experiences; and can occur individually or in groups [11]. A career as a medical practitioner requires more than just clinical reasoning skills and consequently, professional bodies are increasingly emphasizing the importance of engaging in regular reflection, often termed simply as “reflective practice” [12,13]. In medical curricula the skill of reflection is mostly approached through writing or group discussion exercises [14,15]. These activities mostly utilize reflection-on-action through either solitary writing or group communication to improve participants’ understanding and future actions [16].

Reflective writing which is generally completed as a solitary task “shapes the physician’s belief system, guides their practice and nurtures their professional identity formation” [17]. It thereby supports the development of professional skills, standards and expectations, coping and resilience [17], understanding of communication [18], and may improve empathy [8]. Group reflection approaches such as Balint groups and structured group supervision (also known as intervision meetings) also support professional skills and personal development, improve coping and resilience, and can support the modification of behavior. Group approaches can also create safe supportive spaces and grow peer-social cohesion [19,20,21,22]. Despite their potential benefits these two methods have significant limitations with regards to their implementation and alignment with students’ learning preferences. There are students who are comfortable to express their thoughts in writing but would remain silent in group discussions, and students who thrive in a discussion but struggle to express their thoughts in writing. Others will simply have non-linguistic learning preferences [23,24]. Some students will have cultural or linguistic backgrounds which may limit their engagement in written or spoken reflection. While the primacy of speaking and writing as communication methods in society may explain the dominance of written and group reflection as RL approaches, it does not guarantee student engagement. This is illustrated well in a study by Campbell et al. in which many students agreed that ‘writing, reflective, and narrative exercises were a waste of their time’ with the authors noting that these students were also ‘less likely to believe the [group reflective writing] session helped them reflect on clinical experiences’ [25]. Furthermore, there are impactful methodological limitations to written and group discussion approaches. When undertaking reflective writing some students experience anxiety with sharing their thoughts, engage in self-censoring, produce inauthentic reflections, and are disappointed in the lack of personalized feedback received [17]. The benefits of group reflection are also not guaranteed, with benefits to students limited when: trust is not established; facilitation is ineffective; the process, expectations, and outcomes are unclear; participants do not contribute or have negative attitudes or behaviors; or when groups lack sufficient experience or knowledge [20,21,22]. Though the benefits of these approaches are many, their barriers to participation cannot be ignored and alternative or complementary RL methods should be considered. If the reflective approaches offered in a curriculum are not varied then students may not effectively learn and develop a reflective practice, or they may fail to *demonstrate* that they have reflected.

To maximise engagement in reflection, students must be provided with greater diversity of reflective methods from beyond the ‘comfort zone’ of reflective writing and group discussions. Medical curricula should embrace diverse RL methods to enable learners to identify their own personal systems ‘for engaging in reflection’ [26,27], to enhance motivation to participate in reflection, and to support the transition from curriculum directed reflection to lifelong reflective practice [26,28]. Given the potential benefits to students’ wellbeing and burnout reduction from engaging in creative ventures [29,30,31], it is prudent to consider creative approaches to reflective learning. Students are also more likely to engage in tasks that they enjoy and in which they see value, thus providing a range of creative reflective activities to students may heighten their chance of finding a reflective approach that works best for them. Armed with a reflective approach, students could then enter the workforce ready to engage in, and reap the benefits of, reflective practice.

This study explores the literature using thematic analysis to better understand what creative methods of reflective learning have been reported. Reporting upon these creative methods and their learning outcomes will provide guidance for educators in the development of diverse reflective learning models. The research questions for this review were thus: Which types of creative RL methods are described in medical education and what learning outcomes were reported for these methods?

## Methods

We systematically reviewed and thematically synthesized qualitative studies that focus on creative RL in medical education [32]. The Enhancing Transparency in Reporting the Synthesis of Qualitative Research guidelines were utilized to accurately report the methodology of the thematic synthesis [33]. The research was designed with an interpretivist approach to generating and analyzing data within a subjectivist research paradigm.

### Selection criteria

Peer-reviewed papers published in English between 2006-2021 assessing the impact of creative RL approaches on medical students’ development using qualitative or mixed methods designs were included. Earlier publications were excluded as a previous review assessed literature published between 1995–2005 to investigate the utility of reflection and reflective practice in health students’ education [9]. Creative approaches were defined as those not predominantly focused on reflective writing or group discussion as the RL method. Papers were included if they specifically assessed the impact of RL on medical students. Papers focusing only on students’ clinical skills and curriculum knowledge were excluded. Studies without clearly defined RL activities were excluded, as were case-studies, non-primary research articles, meta-syntheses, literature reviews, and systematic reviews.

### Search criteria

A systematic search of Pubmed, PsycINFO, and EMBASE databases was performed in September 2021. Search strategy and results were iteratively developed and reviewed (WM, JP). The search strategy utilized the following search terms in all databases: “reflecti*” and; “medical student*” or “medical edu*” and; “medical school*” or “clinical edu*”. Search syntax was modified for each database with title and abstract fields searched. WM, WC, TP independently screened titles and abstracts. Full-texts of potentially eligible papers were read in entirety to determine eligibility. Reference lists of included articles were hand-searched to identify additional relevant studies. Conflicts were resolved through discussion with another reviewer (JP). Screening results were presented using the Preferred Reporting Items for Systematic Reviews and Meta-Analyses Extension for Scoping Reviews (PRISMA-ScR) statement [34]. The approach to study identification from this systematic review is reported in Supplementary Materials S1.

### Appraisal of included studies

Methodological limitations of included studies were assessed using the Critical Appraisal Skills Programme (CASP) Qualitative Studies Checklist [35]. The domains utilized included aims, methodology, design, recruitment, data collection and analysis, research reflexivity, ethical considerations, and findings. Mixed-methods studies were additionally assessed with the Checklist for Quasi-Experimental Studies [36]. All studies were critically appraised using these checklists (WM). Other reviewers (WC, TP) verified 50% of appraisals. No disagreements in ratings occurred. Appraisal informed judgements about evidence strength and confidence in review findings, but was not used to exclude articles.

### Analysis and synthesis

Previously described methods of thematic analysis and synthesis were used to analyze the selected articles [32,37]. Quotations were extracted from studies’ results/findings sections using line-by-line coding in NVivo Pro software (QRS International, Denver, Colorado). Multiple codes were applied to the same text when deemed appropriate. Discussion sections which included participant quotations were also coded. Initial codes were tested on half the identified literature (n = 7). Further refinements were made iteratively while coding the remaining articles. Sub-themes were derived from the data during an interpretive analysis of perceived benefits to students’ development. Taxonomic analysis identified relationships and similarities between sub-themes, resulting in the construction of themes. Themes from the data were first developed by WM, then validated for relevance and applicability by all authors. Final sub-themes and themes were agreed upon by all authors.

Authors considered the influence of their professional backgrounds, experiences, and prior assumptions during data extraction and analysis. WM has an education and physiology background; WC is a medical student in clinical training; and HW and JP are clinical researchers. WM approached data extraction and theme generation from a perspective free of expectations regarding what education in a clinical setting ‘should’ resemble. WC viewed student quotations from a near-peer perspective and experienced the data more viscerally. HW and JP facilitated discussion of emerging codes and categories with the broader medical education team enabling new insights from those with experience in medical education delivery.

## Results

**Figure 1:**
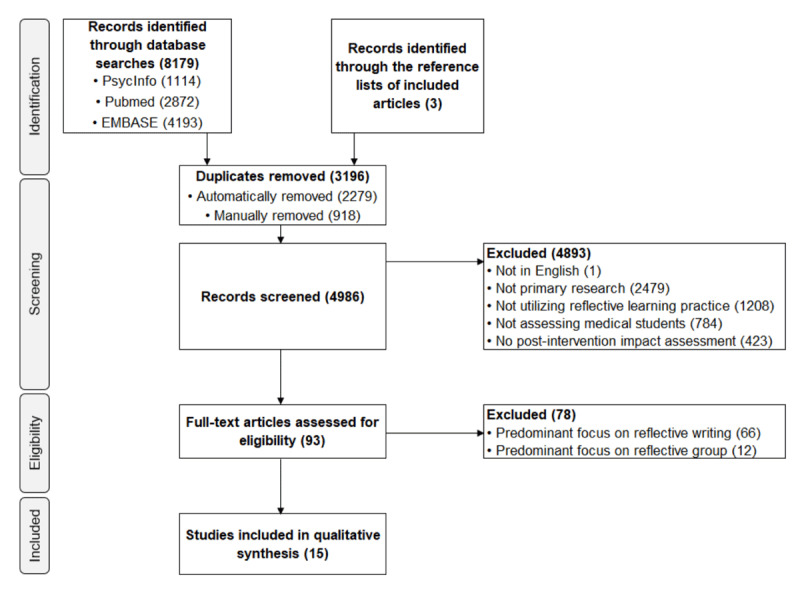
PRISMA-ScR flowchart for the study.

The search process generated 8182 records, with 15 articles eligible for the final synthesis (Figure 1).

### Summary of articles

The included articles represented a geographically diverse range of contexts (Australia, n = 2; Ireland, n = 1; UK, n = 2; USA, n = 7; Lebanon, n = 1; Spain, n = 1, China, n = 1). One article was a pilot study [38] sharing a RL activity that was methodologically similar to another included article by the same first author [39]. Collectively, the studies drew upon over 893 medical students’ perspectives (one study did not provide participant numbers [40]). Study characteristics and appraisal decisions are summarized in Table 1.

**Table 1:** Summary of included articles in a review of Creative Approaches to Reflective Learning amongst Medical Students.


REFERENCE	STUDENT NUMBER	STUDY DESIGN	RLMETHOD	ACTIVITY	REPORTED OUTCOMES	CASP OUTCOME

Brand et al. [38]	10 (4 medical students)	Pre-post survey. Focus groups.	Viewing	Reflection on and discussion of photographs of older persons	Challenged assumptions about ageing and improved Geriatrics Attitudes Scale scores	Minor concerns

Brand et al. [39]	128	Pre-post survey. Written reflections.	Viewing	Reflection on and discussion of photographs of older persons	Challenged assumptions about ageing and improved Geriatrics Attitudes Scale scores	No concerns

Centeno et al. [41]	20	Post survey. Focus groups.	Viewing	Reflection on museum art works	Improved understanding of decision making and individualized care plans	Minor concerns

De la Croix et al. [40]	Not stated	Post survey. Written reflections.	Performing	Learning pre-performative theatre skills	Improved communication, confidence, and provided space for balance, reflection, and understanding	Minor concerns

Gowda et al. [42]	44	Pre-post survey. Focus groups. Written evaluation.	Viewing	Viewing and discussing museum art works	Improved observational skills, awareness of multiple perspectives, a place for restoration and connection with peers	No concerns

Green [43]	42	Pre-post survey.	Viewing	Creating and reading graphic novels	Cultivated a culture of sharing and reflecting upon experiences	Minor concerns

Hayes et al. [44]	10	Reflective diary	Performing	Learning and delivering devised theatre	Developed emotional honesty with peers	Minor concerns

Hoffman et al. [45]	18	End of course evaluation	Performing	Learning improvisational theatre	Improved active listening and appreciation of the perspectives of others	Minor concerns

Neel et al. [46]	21	Pre-post survey	Performing	Learning improvisational theatre	Improved proactivity, wellbeing, and communication	Moderate concerns

Osman et al. [47]	42	Written reflections	Imagining	Prioritizing end of life decisions	Developed recognition of differing priorities and highlighted the importance of personalized care	Moderate concerns

Pacala et al. [48]	477	End of course evaluation	Imagining	Role playing the ageing process	Improved attitudes towards aging and understanding of older adults’ experiences and perspectives	Minor concerns

Potash et al. [49]	20	Pre-post survey	Creating	Art making (poem and artwork)	Increased emotional awareness and self-understanding	Minor concerns

Sandars et al. [50]	12	Focus group	Creating	Creating digital storytelling videos	Encouraged deeper reflection	Minor concerns

Saunders et al. [51]	82	Open-ended reflection questions	Mind-body	Mind-body skills course	Built connections with peers, increased self-awareness, and changed attitudes towards medical school	Minor concerns

Shapiro et al. [52]	25	Group interview. Written feedback.	Viewing	Viewing and discussing art or dance	Increased awareness of emotional responses in self and others and helped students see more depth in patients	Minor concerns


Abbreviations: CASP = Critical Appraisal Skills Programme; RL = Reflective Learning.

### Reflective methods

Methods to stimulate RL were organized into five categories: viewing, performing, creating, imagining, and mind-body (Table 1).

*Viewing* utilized visual stimulus items as reflective prompts. Some studies reflected-in-action utilizing learning activities [42] or facilitated discussions [52] to investigate artworks. Others reflected-on-action with informal or facilitated discussions after viewing photography [38,39] or artworks [41], or utilized private reading of graphic medicine texts [43].

*Performing* taught performance exercises to develop skills relevant to medical practice such as active listing and recognizing bias. One study taught pre-performative skills (e.g., voice skills) [40], two utilized improvisational theatre activities [45,46], and one culminated in a devised theatre performance to peers [44].

*Creating* emphasized reflection-on-action through creating either medical comics [43]; poetry, drawings or paintings [49], or using digital storytelling [50].

*Imagining* studies focused on role-playing medicine through the patient lens. One study utilized live-action role-play with students as older adults with physical limitations [48]. The other involved prioritizing 36 end-of-life choices to facilitate consideration of patient perspectives [47].

*Mind-Body* included one study teaching skills such as relaxation techniques and meditation [51].

### Thematic synthesis

**Figure 2:**
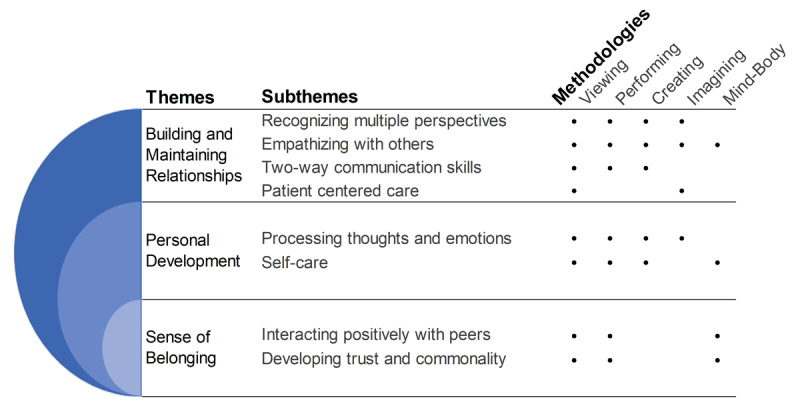
A model of the effects of creative reflective learning on medical students’ development.

Coding generated rich data describing multi-faceted student development in response to creative RL. Generated themes describe personal development, building and maintaining relationships, and sense of belonging. Themes, sub-themes and methods are shown in Figure 2.

### Personal development

#### Processing thoughts and emotions

Creative RL provided opportunities for exploring thoughts and emotions. Students identified ‘disconnects’ between their ‘intentions, feelings, and actions’ [51]. Some formed ‘different connections’ with themselves [41] and better understood their emotional triggers or were able to realize that their experiences actually affected them [50]. Students’ sentiments towards emotions [44,47] and abilities to recognize their internalized biases also improved [39,40,42,43,47,48].

#### Self-care

RL was a therapeutic curriculum break described as a: ‘sanctuary’ [42], ‘reset button’ [46], ‘chance to escape … and relax’ [49], and an activity you would ‘want to do’ [50]. Participation increased students’ attention to self-care activities and facilitated uptake of relaxing past-times [42,51]. RL also normalized self-care among students ‘It’s healthy to do that (self-care). It will make me a better doctor to do that’ [42]. Mind-body skills equipped students with a ‘new set of tools’ for managing life and medical school stresses: ‘I listen to my body more. I’m more attentive to my state of mind. I feel that I have more control over myself’ [51]. Similarly, performing arts activities helped students to identify and modify ‘the physical effects of stress’ [40] and normalized failure [46].

### Building and maintaining relationships

#### Recognizing multiple perspectives

Students identified areas where their perspectives differed from peers. For instance, their interpretations of portraiture [39] and emotions [42], and views on sexuality [40], art [42,52], and end-of-life priorities [47]. The encountered perspectives surprised some students [38,39,40,49]: ‘… there was so much room for imagination and creativity when thinking of a person, patient or illness’ [49]. Recognizing other perspectives engendered empathy towards patients in some students. ‘…[This game] has given me more empathy for my elderly patients’ [48].

#### Empathizing with others

Students had more empathy for patients following RL activities [38,48,52]. ‘… [I] think of my own back all hunched over and I felt like I was in pain … my immediate response was she’s probably in pain’ [38]. Students demonstrated empathic imagination by juxtaposing artistic pieces with real situations [40,41,49], imagining patients’ perspectives through role-play [48], discussion of photography [38], reading or creating comics [43], and prioritizing end-of-life decisions [47]. Students subsequently intended to: provide ‘sensitive’ care [41] and doctor-patient ‘partnerships’ [51], soften ‘hard edges for vulnerable patients’ [40], and be a support person to patients [41,49]. This shows students’ growing desires to embed empathy in their professional identities. ‘… you have to be very sensitive to what happens around you and that art helps to see and promote that sensitivity…’ [41].

#### Two-way communication skills

Students noted RL helped develop effective communication, increased awareness of ‘their own bodies and physical responses’ and the ‘physicality of others’ [40], and highlighted the value of non-verbal communication skills. ‘…often times they [non-verbal communication] can tell you more than verbal language would’ [46]. Students reading graphic medicine texts also noted improved awareness and understanding of non-verbal cues and communication [43].

#### Patient-centered care

Several studies showed students intended to incorporate patient-centered care practices into their professional identity by recognizing patients as individuals requiring individualized care. Students found ‘one-size fits all’ approaches inappropriate [39], as all patients have ‘other things in their life’ [41], ‘different priorities and values’ [47], and that doctors are not always the ‘center of care’ for their patients [43]. Awareness of influences on patients may thus engender patient-centered care intentions. ‘I should stop seeing patients like machines to [be] fix[ed] and start seeing patients like persons who deserve a better way of life’ [41].

### A sense of belonging

#### Interacting positively with peers

RL provided opportunities to interact with classmates in ‘positive, low-stress setting[s]’ [46]. Peer interactions outside the standard medical curriculum facilitated the formation of social connections and friendships [40,42,44,46,51].

#### Developing trust and commonality

Students built trust [40], improved teamwork skills [40,44], shared their thoughts, feelings and experiences with others [40,42,44,51], and identified that their ‘core worries and niggles’ about medical school were common [40,51]. ‘… I’m not alone in my fears to succeed in med school…’ [51].

## Discussion

This synthesis of qualitative and mixed methods studies analyzed literature reporting on studies using creatively focused RL. Our findings indicate that creative RL methods afford students the opportunity to experience and engage in diverse reflective practices whilst providing alternate learning approaches to those who do not benefit from conventional RL methods such as written and group approaches. With our findings, we aim to provide evidence-based recommendations for the development of meaningful and flexible reflective learning models.

Creative RL can embrace diversity in reflective practice and facilitate individual approaches for learners, thereby facilitating true reflection [28]. However, ensuring students complete reflective exercises is not a guarantee that they have reflected as reflective behaviors can be inauthentic [17,26]. RL activities should therefore be designed to make students *want* to participate in reflection. This may be accomplished by accepting and embracing diversity, encouraging universities to try different models of reflection, and by creating conditions which foster reflection [26,28]. The reflective approaches currently utilized in medical education tend to emphasize reflection based upon written and verbal approaches, perhaps reflecting a societal disposition towards written and spoken communication models. Creative RL methods are uniquely placed to boost student participation and interest in reflective learning as they offer diverse methods of engagement and (due to their interpretivist nature) opportunities to develop reflective capacity without ones’ views being deemed right or wrong.

This study offers practical information for those implementing RL programs in medical curricula by outlining what creative approaches to RL have been implemented and their outcomes. Based on benefits reported in the included studies three key themes were developed, personal development, building and maintaining relationships, and sense of belonging. The studies underpinning these themes were further organized by RL method into the categories viewing, performing, creating, imagining, and mind-body. The categories themselves speak to the breadth of approaches which could be taken beyond the common choices of reflective writing and group discussions. Though the reported benefits of RL varied there were several benefits which appeared across the majority of RL methods. Namely, improvements in empathizing with others, recognizing multiple perspectives, processing thoughts and emotions, and self-care. Other potential benefits present in some methods included improved two-way communication skills, patient centered care, positive interactions with others, and developing trust and commonality.

A comparison of the benefits of creative RL versus written and group RL indicates many similarities. Written and creative RL have evidence of improving patient care, self-monitoring, and self-improvement. Creative and group RL approaches report improved personal and professional development, sense of belonging or social-cohesion, trust and safety within the peer-group, understanding of self and others, self-confidence, improved communication, and modifications to participants behaviors and habits. Creative, written, and group approaches refer to improvements in recognizing and valuing other perspectives, developing empathy, and improving coping skills. Naturally, the limitations of implementing creative RL practices must be considered alongside the potential benefits. The studies included in this review mentioned few limitations beyond sparing references to the time pressures of including RL activities in the curriculum [41] and that some students may be initially skeptical of arts-based teaching [40]. Given the optional nature of most of the creative RL interventions, selection bias may partly explain the lack of detail on limitations. Drawing upon the literature for group and written RL provides some initial guidance on how to avoid potential barriers to learning when implementing creative RL approaches. For instance, the purpose of reflection should be clear; facilitators should be trained, confident, and committed; reflection must be role modelled; and the structure of reflection should be safe, flexible, and creative [28]. Discussions should be confidential; facilitators competent and willing to show vulnerability; and personalized student feedback should be provided [17,18,20,28].

The definition of “creative approaches” applied during the literature review is subjective. This may have resulted in the exclusion of some articles which others may have included, however researchers of diverse backgrounds contributed to literature screening and data analysis to limit the impact of researcher bias. Some studies were excluded as they did not articulate the benefits of their RL approaches to students, despite using creative RL approaches. We also did not contact medical schools about potentially novel unpublished RL practices. We believe that the addition of further articles and RL methods would not necessarily change synthesis findings, as high levels of repetition in some themes suggests that saturation was approached, if not reached. This study forwent the exploration of all potential creative RL approaches and instead provides an evidence-based approach to the benefits to students thereby providing a valuable resource which medical educators can refer to when reviewing RL practices and seeking evidence to justify change.

The findings of this study illustrate the types of creative RL approaches which could be used in medical curricula and highlight how they may benefit students. It was shown that creative RL approaches can foster medical students’ sense of belonging, interpersonal skills, and personal development and have considerable potential to holistically improve overall medical student development. The relationship between creative RL and students’ sense of belonging warrants further investigation as stronger support networks are known to assist in managing emotional stresses. When reviewing medical curricula, educators should pause to consider whether creative approaches to reflective learning could provide students with opportunities to develop their personal reflective style, develop as reflective practitioners, and support student needs such as sense of community or self-care behaviors. We believe our findings will embolden educators to utilize greater diversity of reflective learning approaches to support the needs of medical students whilst helping them develop their own unique approach to reflective practice.
